# Decreasing Barriers to Care: Voices of Riders, Drivers, and Staff of a Rural Transportation Program

**DOI:** 10.1093/geroni/igab046.2861

**Published:** 2021-12-17

**Authors:** Abby Schwartz, Alice Richman, Mallary Scott, Haiyong Liu, Weyling White, Caroline Doherty

**Affiliations:** 1 East Carolina University, School of Social Work, Greenville, North Carolina, United States; 2 East Carolina University, East Carolina University, North Carolina, United States; 3 East Carolina University, Greenville, North Carolina, United States; 4 Roanoke Chowan Community Health Center, Ahoskie, North Carolina, United States

## Abstract

Eastern North Carolina (eNC) is a rural, poor, and underserved region of the state with 1 in 5 adults living below the poverty level. Residents experience health disparities driven by limited access to healthcare and inequitable distribution of social determinants of health. Project TRIP (Transporting Residents with Innovative Practices) is a potential solution to barriers in accessing care in eNC. Results presented include the first phase of a multi-phase study evaluating and replicating TRIP’s effectiveness. Data from qualitative interviews with TRIP riders, drivers, and staff (e.g., case managers) will be presented (n= 20). As a result of the COVID-19 pandemic, interviews were conducted by telephone with the goal of understanding both strengths and weaknesses of the transportation program from riders, drivers, and staff to gain a holistic understanding of TRIP. Of the riders interviewed, the majority (91%) were age 50 and over and African American. Themes that emerged from the data that highlighted strengths of the program included: improved health outcomes, no wait times for pick up or drop offs, cost free, and accommodating service. Themes related to areas of weaknesses or improvement included: needing more transportation vendors and a dedicated TRIP case manager and scheduling concerns. The presentation will conclude with considerations in translating the findings into a pilot and expansion of TRIP in another eNC county (study phases 2 & 3), and how the data can inform the development of transportation interventions in other states, with the goal of increasing access to healthcare for vulnerable rural populations.

